# The potential applications of Apolipoprotein E in personalized medicine

**DOI:** 10.3389/fnagi.2014.00154

**Published:** 2014-07-08

**Authors:** Sylvia Villeneuve, Diane Brisson, Natalie L. Marchant, Daniel Gaudet

**Affiliations:** ^1^Department of Medicine, ECOGENE-21 and Lipid Clinic, Chicoutimi Hospital, Université de MontréalChicoutimi, QC, Canada; ^2^Helen Wills Neuroscience Institute, University of CaliforniaBerkeley, CA, USA; ^3^Department of Old Age Psychiatry, Institute of Psychiatry, King's College LondonLondon, UK

**Keywords:** ApoE, cardiovascular diseases, Alzheimer disease, neurodegenerative diseases, risk, diagnosis, prognosis, treatment

## Abstract

Personalized medicine uses various individual characteristics to guide medical decisions. Apolipoprotein (ApoE), the most studied polymorphism in humans, has been associated with several diseases. The purpose of this review is to elucidate the potential role of ApoE polymorphisms in personalized medicine, with a specific focus on neurodegenerative diseases, by giving an overview of its influence on disease risk assessment, diagnosis, prognosis, and therapy. This review is not a systematic inventory of the literature, but rather a summary and discussion of novel, influential and promising works in the field of ApoE research that could be valuable for personalized medicine. Empirical evidence suggests that ApoE genotype informs pre-symptomatic risk for a wide variety of diseases, is valuable for the diagnosis of type III dysbetalipoproteinemia, increases risk of dementia in neurodegenerative diseases, and is associated with a poor prognosis following acute brain damage. ApoE status appears to influence the efficacy of certain drugs, outcome of clinical trials, and might also give insight into disease prevention. Assessing ApoE genotype might therefore help to guide medical decisions in clinical practice.

## Introduction

### Personalized medicine and genetic

Personalized medicine refers to medical care that uses individual characteristics, such as family and personal history, gene, protein, or environmental information to facilitate a global analysis of individual risk evaluation, diagnosis and prognosis, and improve therapeutic and preventive approaches for patients (Offit, 2011). Personalized medicine would therefore guide medical decisions. Even though the practice of personalized medicine is not new, there is an increasing enthusiasm in this approach which can be attributed to the exploding field of genetic research.

Apolipoprotein (ApoE) is a protein that is well known for its key roles in the transport of cholesterol and other lipids in blood circulation and central nervous system (CNS). Since its identification in 1973 (Shore and Shore, 1973), ApoE has become one of the most widely studied gene variants, not only for cardiovascular disorders but also in relation to several other medical conditions such as neurodegenerative and autoimmune diseases (Mahley, 1988; Mahley et al., 2009; Verghese et al., 2011). Given its broad biological role, ApoE may therefore become a major variable of preventive medicine. This review aims to summarize the role of ApoE in components of personalized medicine, including the individual risk prediction, diagnosis, prognosis, and treatment of associated diseases. This review is intended to summarize and discuss novel, influential and promising works in the field with a specific, but not exclusive, focus on neurodegenerative diseases.

## Apolipoprotein E basic structures and function

ApoE includes three common isoforms (ApoE2, ApoE3, ApoE4), coded by three codominant alleles (e2, e3, e4). Hence, six phenotypes commonly exist in the general population, three homozygous (ApoE2/2, ApoE3/3, and ApoE4/4) and three heterozygous (ApoE2/3, ApoE2/4, and ApoE3/4). ApoE2 and ApoE4 differ from ApoE3 by a single amino acid substitution. While ApoE3 contains cysteine at position 112 and arginine at position 158, the arginine is substituted by a cysteine in ApoE2 carriers and the cysteine is substituted by an arginine in ApoE4 carriers (Weisgraber et al., 1982; Mahley, 1988). This arginine substitution results in an interaction between ApoE4 amino- and carboxyl-terminal domains, known as “domain interaction” (Mahley and Huang, 2009). The structural difference due to domain interaction is believed to play a role in the altered function of ApoeE4.

ApoE is synthesized predominantly in the liver but is also found in the brain, spleen, lung, kidney, ovary, testis, peripheral nerves and muscular tissues (Zhang et al., 2011). In the brain, ApoE is produced by astrocytes and, to a minor extent, by microglia (Pitas et al., 1987; Grehan et al., 2001), and can also be produced by neurons in response to neuronal injury or stress (Mahley and Huang, 2012a). ApoE's principal known function is to transport cholesterol and other lipids in blood circulation and the CNS by binding to ApoE receptors on their cell surface (Mahley, 1988). ApoE is also essential to maintain the structural integrity of lipoproteins, stabilize, and solubilize liproproteins in the blood, and to serve as cofactors in enzymatic reaction (Eichner et al., 2002). In the brain, cholesterol plays a crucial role in myelin production and is an essential component of membrane sheaths, brain development, neuronal maintenance, neuronal repair, and long-term synaptic plasticity (Mauch et al., 2001; Bu, 2009; Mahley et al., 2009; Leduc et al., 2011).

## Risk assessment

Because of its key role in the regulation of lipid metabolism ApoE was first recognized for its role in cardiovascular diseases (CVD). While the ApoE3 isoform is not associated with an increased risk of CVD, the ApoE4 isoform is a major risk factor for CVD, such as coronary heart disease, arrhythmias, and stroke (Lahoz et al., 2001; Eichner et al., 2002; Song et al., 2004; Mahley et al., 2009). ApoE2 has a more ambiguous relationship with CVD as it has been associated with both an increased (Mahley et al., 1999; Lahoz et al., 2001) and reduced (Bennet et al., 2007) risk of CVD. This duality found in the ApoE2 isoform suggests that the adverse or beneficial impact of ApoE2, and potentially ApoE4, on disease risk is influenced by it interaction with other genetic or environmental factors (Lahoz et al., 2001; Elosua et al., 2003; Mahley et al., 2009).

It is now well recognized that ApoE is also associated with a variety of other diseases. ApoE4 for instance is the most important genetic risk factor for late onset Alzheimer Disease (AD) (Poirier et al., 1993; Leduc et al., 2011; Verghese et al., 2011) with ApoE4 carriers having a 4 to 12-fold increased risk of developing AD when compared to non-carriers (Corder et al., 1993, 1994; Bertram and Tanzi, 2008; Mahley and Huang, 2012a). The ApoE2 isoform on the other hand is protective against AD (Corder et al., 1993, 1994). How these isoforms differently affect the risk of AD is not fully understood. Several mechanisms have been implicated. One potential mechanism is via accumulation and/or clearance of amyloid-β (Aβ, a hallmark of AD) from the brain because ApoE4 has been associated with greater Aβ burden (Strittmatter et al., 1993; Ye et al., 2005; Drzezga et al., 2009) and ApoE2 with lower Aβ burden (Jiang et al., 2008; Morris et al., 2010). It is well established that ApoE binds directly to the Aβ peptide. Compared to the other isoforms, ApoE4 binds to Aβ with lower affinity, which might result in less efficient clearing of Aβ through the cell surface (Ladu et al., 1994; Yang et al., 1999; Tokuda et al., 2000). In the same line, ApoE4 is associated with slower transport of Aβ across the blood-brain barrier (BBB), causing greater Aβ retention in mice brains (Deane et al., 2008). ApoE4 is also associated with BBB dysfunction, which might lead to Aβ accumulation, and also to a reduction in cerebral blood flow and hypoxia, two conditions that enhance brain vulnerability (Zlokovic, 2011). While several routes exist for the clearance of Aβ, clearance directly into the blood seems to be the predominant pathway in the human brain (Bell and Zlokovic, 2009; Bell et al., 2012). Accordingly, vessel wall thickness and reduced cerebral blood flow may result in Aβ accumulation. Supporting this idea, ApoE4 carriers are known to have higher accumulation of Aβ in cerebral blood vessels, a condition known as cerebral amyloid angiopathy (CAA) (Verghese et al., 2011). ApoE4 is also associated with greater neuronal inflammation (Grainger et al., 2004; Guo et al., 2004; Kim et al., 2009; Leduc et al., 2011) and less efficient neuronal repair (Mauch et al., 2001; Bu, 2009; Mahley et al., 2009; Leduc et al., 2011), two other conditions that might influence the risk of AD. Finally, in response to neuronal injury, neuron-ApoE4 production can generate neurotoxic fragments that exacerbate neuronal toxicity, increase tau phosphorylation, NFT formation, neuronal mitochondrial dysfunction, and decrease GABAergic interneuron selectivity (Mahley and Huang, 2012a; Liu et al., 2013).

The association of ApoE with other neurodegenerative diseases is less clear. Recent meta analyses suggest that ApoE4 carriers might be more susceptible to Creutzfeldt–Jakob disease (Wei et al., 2014). ApoE2 in turn might increase the risk of age-related macular degeneration (Leduc et al., 2011) and Parkinson disease (Huang et al., 2004) highlighting again the ambiguous role of ApoE2.

As it can be seen in Table 1, the impact of ApoE goes far beyond the boundary of CVD and neurodegenerative diseases. Links have been suggested for instance between ApoE4 and the susceptibility to malaria, viral infections such as herpes simplex virus (HSV), human immunodeficiency virus (HIV) (Mahley et al., 2009; Zhang et al., 2011), cancer (Porrata-Doria et al., 2010; Kulminski et al., 2011; Saadat, 2012), and gallbladder stone disease (Xue et al., 2012). Even if these last associations are not always consistent between studies, all together they suggest that ApoE influences the risk of a variety of diseases which supports the idea that ApoE influences human health via multiple pathways.

**Table 1:** **Association of ApoE to pre-symptomatic risk, prognosis, and response to treatment**.

	**Phenotype**	**Risk**	**Prognosis**	**Treatment**
**HYPERLIPIDEMIA (Mahley, 1988; Mahley et al., 1999; Maitland-Van Der Zee et al., 2003; Nieminen et al., 2008; Dergunov, 2011)**				
Familial hyperlipidemia and hypercholesterolemia	E2	(−)		Highest response to lipid-lowering therapy
	E4	+		Lowest response to lipid-lowering therapy
				Lowest compliance to treatment
				Highest response to low fat diet
Familial dysbetalipoproteinemia	E2	+		
	E4			
**CARDIOVASCULAR DISEASES (Lahoz et al., 2001; Eichner et al., 2002; Masson et al., 2003; Song et al., 2004; Martinez-Gonzalez and Sudlow, 2006; Stehle et al., 2008; Mahley et al., 2009; Guo et al., 2011; Khan et al., 2013; Rannikmae et al., 2013)**				
Stroke	E2			
	E4	(+)	Worst prognosis	
			↑ Risk of death^*^	
Coronary heart disease	E2	(−)		
	E4	+	↑ Risk of death	
Cerebral amyloid angiopathy (CAA)	E2		↑ Risk hemorrhage	
	E4	+	↑ Risk hemorrhage	
			↑ Severity	
			↑ Risk death	
**NEURODEGENERATIVE DISEASES (Poirier et al., 1993; De La Fuente-Fernandez et al., 1999; Bedlack et al., 2000; Leduc et al., 2011; Verghese et al., 2011; Panza et al., 2012; Yin et al., 2012)**				
Alzheimer disease (AD)	E2	−	Later onset	
	E4	+	Earlier onset	Cognition: Lowest response or no improvement in persons with dementia but higher response in persons with mild cognitive impairment Biomarkers (amyloid and CSF tau): higher response in persons with dementia
			↑ Neuropsychiatric symptoms	
Vascular disease-associated cognitive impairment	E2			
	E4		↑ Risk dementia	
Lewy body disease	E2			
	E4	(+)	↑ Risk dementia	
Pick's disease	E2			
	E4		Earlier onset	
			↑ Risk dementia	
Corticobasal degeneration	E2			
	E4		↑ Risk dementia	
Down'syndrome	E2			
	E4		Faster progression	
			↑ Risk dementia	
Parkinson's disease (PD)	E2	+		
			↑ Risk dementia	E4/4: ↑ Drug-induced hallucination
Amyotrophic lateral sclerosis (ALS)	E2		Later onset	
	E4		Earlier onset	
			Worst prognosis	
Age-related macular degeneration	E2	(+)	Earlier onset	
	E4	(−)	Later onset	
**AUTOIMMUNE DISEASES (Corder et al., 1998; Roselaar and Daugherty, 1998; De Bont et al., 1999; Mahley et al., 2009; Vitek et al., 2009; Kuhlmann et al., 2010; Zhang et al., 2011)**				
Human immunodeficiency virus (HIV)	E2			(↑ Risk hypertriglyceridemia)
	E4	(+)	↑ Risk dementia	(↑ Risk hypertriglyceridemia)
			↑ Risk Peripheral neuropathy	
			↑ Risk AIDS	
Acquired immunodeficiency syndrome (AIDS)	E2			
	E4	+	Worst prognosis	
Hepatitis C virus	E2		E3 and E2: ↑ Risk chronic infection	
	E4			(Lowest response to antiviral therapy)
**OTHER [TRAUMATIC BRAIN INJURY (Lichtman et al., 2000; Chamelian et al., 2004; Crawford et al., 2009; Hiekkanen et al., 2009; Ponsford et al., 2011); DIABETES MELLITUS (Tsuzuki et al., 1998; Bedlack et al., 2003; Saito et al., 2004; Yin et al., 2013); CANCER (Ahles et al., 2003; Porrata-Doria et al., 2010; Kulminski et al., 2011; Saadat, 2012); DEPRESSION (Irie et al., 2008; Kim et al., 2010, 2011; Meng and D'Arcy, 2013)]**				
Traumatic brain injury	E2			
	E4		Worst prognosis	
Diabetes mellitus	E2			
	E4		↑ Risk diabetic neuropathy	Best response to low fat diet
Cancer	E2			
	E4	(+)		(E4: ↑ side effect following chemotherapy)
Depression	E2			
	E4		↑ Risk dementia	

Creutzfeldt-Jakob disease, gallbladder stone disease, Huntington's disease, inclusion-body myositis, cerebral palsy, progressive supranuclear palsy, temporal lobe epilepsy multiple system atrophy, multiple sclerosis, frontotemporal dementia, malaria and herpes simplex virus need further exploration (Bu, 2009; Leduc et al., 2011; Verghese et al., 2011; Xue et al., 2012; Yin et al., 2012; Rubino et al., 2013; Wei et al., 2014).

* In subarachnoid hemorrhage and intracerebral hemorrhage stroke;

+, increased risk; −, decreased risk; (parentheses), limited, or conflicting results exist.

At present the utility of determining ApoE genotype in everyday practice is questionable for two reasons: ApoE is principally a risk factor and not a causal factor, and inconsistencies remain in the literature, particularly for diseases other than CVD and AD. Understanding the cause of these inconsistencies, or why some persons do not develop a specific disease, might however yield great potential for disease prevention. Indeed, for some diseases, ApoE might only have a detrimental effect under specific circumstances or when interacting with other factors. While ApoE alone might therefore not be useful to assess risk of developing a specific disease, it could be included in an integrated risk evaluation metric. The association between the e4 allele and a poor vascular health for instance seems to be stronger in the presence of life stress factors and habits such as obesity, alcohol consumption, and smoking (Corella et al., 2001; Djousse et al., 2002; Zeng et al., 2011). Indeed weight gain and obesity appear to have a particularly severe effect on ApoE4 carriers, and have been associated with increases in triglycerides, β lipoprotein (Gueguen et al., 1989), total insulin and LDL cholesterol levels (Marques-Vidal et al., 2003). If clinicians possessed this knowledge and ApoE genotype information, they could provide more patient-specific treatment advice that would particularly help ApoE4 carriers reduce their risk of CVD. Another example would be the interaction between ApoE and lifestyle on the risk of AD. Increased physical activities and good vascular health appear to reduce the negative effect of ApoE4 on Aβ and AD (Niti et al., 2008; Ferrari et al., 2013). Physical activity also reduces the rate of hippocampal atrophy in ApoE4 carriers, an effect that was not observed in non-carriers (Smith et al., 2014). The hippocampus is a key structure for episodic memory formation (Villeneuve and Belleville, 2012), and is particularly sensitive to AD. Lifetime cognitive activity also seems to reduce the negative impact of ApoE on Aβ burden, since ApoE4 carriers that report high lifetime cognitive activity have similar Aβ brain burden than non-carriers (Wirth et al., 2014). Incorporating these findings into medical practice would suggest that physical and cognitive activity could be used as preventive activities to reduce the risk of AD in ApoE4 carriers. With no current treatment for AD, and with ApoE being the main risk factor for this disease, better understanding of the factors that interact with ApoE-related risk to postpone the development of the disease is needed given their potential implication in preventive medicine. Understanding these interactions might also be valuable to guide new treatment developments.

## Diagnosis

As mentioned previously, although knowing ApoE status is valuable to assess pre-symptomatic risk for disease, it is rarely sufficient to make a diagnosis. Type III dysbetalipoproteinemia diagnosis, a rare inherited disorder caused by ApoE (commonly ApoE2) defective binding to lipoprotein receptor, is however an exception (Mahley et al., 1999). Type III is mainly—but not exclusively—diagnosed among individuals who are homozygous for the apolipoprotein e2 allele (ApoE2/2). Therefore, assessment of ApoE genotype plays a critical role in type III dysbetalipoproteinemia diagnosis. However, only a minority of ApoE2/2 carriers develops this disease. Furthermore, even in subjects who develop the disease, the majority remain normolipidemic for decades, particularly in the absence of additional primary or secondary dyslipidemic factors. This last point supports the value of knowing the ApoE status in patients with a family history of type III dysbetalipoproteinemia because by controlling, or preventing, other dyslipidemic factors it is may be possible to postpone the expression of the disease.

## Prognosis

In addition to affecting the risk for several diseases, ApoE can influence the pattern of their progression. For instance in several types of dementia, ApoE4 has been related to earlier onset as well as higher risk of neuropsychiatric symptoms (Leduc et al., 2011; Panza et al., 2012). An obvious example is in AD were ApoE4 carriers develop dementia 8–20 years earlier then non-carriers (Corder et al., 1993; Mahley and Huang, 2012a). ApoE4 is also associated with an increased risk of dementia in Parkinson disease, Lewy body disease, Pick's disease, vascular diseases, Down's syndrome, corticobasal degeneration, and HIV (Mahley et al., 2009; Leduc et al., 2011; Verghese et al., 2011). While ApoeE4 does not appear to increase risk for depression *per se* (Mauricio et al., 2000; Blazer et al., 2002), it has been associated with an increased risk for dementia in individuals with depression (Irie et al., 2008; Kim et al., 2010, 2011; Meng and D'Arcy, 2013). Except for AD, these findings suggest that ApoE might not be associated with the disease pathology itself, but that ApoE4 makes the brain less resiliant to neurodegenerative processes. Knowing the ApoE genotype of a person with a neurodegenerative disease might therefore give insight on the risk of developing dementia. Regular cognitive evaluations might be appropriate in such cases.

The interaction between neurodegenerative diseases such as AD and ApoE genotype on rate of cognitive decline have been extensively studied, but the findings are inconsistant. While some studies suggest that ApoE4 AD carriers have a faster rate of cognitive decline (Craft et al., 1998; Martins et al., 2005; Schmidt et al., 2012), others suggest no association (Growdon et al., 1996; Kleiman et al., 2006). It has even been proposed that non-carriers present a slightly different, and more agressive, form of AD and that the latter group shows a faster progression than the ApoE4 carriers (Stern et al., 1997). In regard to persons with mild cognitive impairment, the presence of an e4 allele is associated with an increased risk of progression to dementia (Mosconi et al., 2004; Petersen et al., 2005; Vos et al., 2013). In such cases, knowing the ApoE status seems to give useful information about the prognosis. Because mild cognitive impairment can be caused by several conditions such as AD, vascular disease, Parkinson disease, and depression (Gauthier et al., 2006; Villeneuve et al., 2011a,b, 2012), knowing the ApoE status could also give insight about the source of disease. Indeed, as mentioned previously ApoE4 is the strongest genetic risk factor for AD and ApoE4 carriers often have higher Aβ burden. Accordingly, ApoE4 carriers with mild cognitive impairment are usually considered at increased risk of AD (Petersen et al., 2005).

Knowledge of ApoE genotype also provides valuable information about recovery prognosis after an acute event such as stroke (Martinez-Gonzalez and Sudlow, 2006; Guo et al., 2011), delirium (Adamis et al., 2007), or traumatic brain injury (TBI) (Lichtman et al., 2000; Crawford et al., 2009; Mahley et al., 2009; Ponsford et al., 2011). In all these cases, ApoE4 carriers show a worse long-term outcome than ApoE3 carriers, and even sometimes higher risk of death. The presence of an e4 allele has also been associated with greater cognitive decline in older stroke patients (Ballard et al., 2004). A predominant hypothesis in the literature is that this poor outcome is caused by a lack of reparative capacity of ApoE4 (Crawford et al., 2009; Ponsford et al., 2011). Given this knowledge, after an adverse event ApoE4 carriers should have a closer follow-up and older adults should be assessed for associated cognitive deficits.

The value of knowing the ApoE status in the prediction of disease prognosis is not restricted to neurodegenerative diseases or brain integrity as can be seen in Table 1. ApoE4 patients affected by HIV are for instance at higher risk of developing peripheral neuropathy and acquired immunodeficiency syndrome (AIDS). Higher cerebrospinal fluid (CSF) ApoE levels have also been associated with higher cognitive impairment in patients affected by HIV (Andres et al., 2011), a finding that was also present in persons with mild cognitive impairment (Song et al., 2012) but not in AD patients (Schmidt et al., 2014). In addition, ApoE4/4 carriers suffering from AIDS have accelerated disease progression and increased risk of death (Corder et al., 1998; Zhang et al., 2011). These data suggest that, in regard to many diseases, a closer follow-up is needed in ApoE4 carriers since this genotype is associated with worst prognosis.

## Treatment

### ApoE and dose response

It is well known that genetics influence drug absorption, distribution, and metabolism, leading to differences in dose responses and side effects. Several reports suggest that ApoE alleles influence therapy efficiency for dyslipidemias (Brisson et al., 2002; Nieminen et al., 2008; Dergunov, 2011). In fact, ApoE genotype is considered the most significant predictor of lipid response to statins and fibrates, two classes of lipid-lowering therapy which act via different mechanisms (Dergunov, 2011). While individuals with ApoE4 seem to have the poorest response to lipid-lowering therapy, individuals with ApoE2 have the highest response (Dergunov, 2011). This result has been shown using different molecules (Ordovas et al., 1995; Nestel et al., 1997; Sanllehy et al., 1998; Ballantyne et al., 2000; Pedro-Botet et al., 2001; Zuccaro et al., 2007) and has been replicated in familial hypercholesterolemia patients (O'Malley and Illingworth, 1990; Carmena et al., 1993). Phenotypic differences in lipid metabolism may help explain these findings (Weisgraber et al., 1982; Mahley, 1988; Dong et al., 1994; Dong and Weisgraber, 1996; Petersen et al., 2005; Hatters et al., 2006).

Assessing ApoE polymorphism might be particularly important in clinical trials. In a Phase IIB clinical trial of an anti-diabetic drug, rosiglitazone, to target mild to moderate AD patients, the results were inconclusive when patients with and without ApoE4 were pooled together (Risner et al., 2006). However, when ApoE4 carriers were subtracted from the analysis, a significant cognitive improvement was observed in ApoE4 non-carriers (Risner et al., 2006). A recent clinical trial of Bapineuzumab, an anti-amyloidβ monoclonal antibody, conducted in mild to moderate AD patients reported reduction in cerebral Aβ and CSF phospho-tau concentrations in ApoE4 carriers, but not in non-carriers (Salloway et al., 2014). Turning to the cholinergic system, response profiles of ApoE4 carriers have been mixed in AD patients with dementia (Poirier et al., 1995; Farlow et al., 1996, 1998; Bizzarro et al., 2005; Choi et al., 2008; Carriere et al., 2009), but non-demented ApoE4 carriers have shown greater cognitive response to the cholinesterase inhibitor, donepezil (Petersen et al., 2005), and to the cholinergic agonist, nicotine (Marchant et al., 2010; Evans et al., 2013). The severity of the disease might therefore also be an important factor to consider when assessing these relationships.

The effect of ApoE on response to treatment in other diseases is not clear and/or research is lacking (Stehle et al., 2008; Carmona et al., 2011; Guerrero et al., 2011). One interseting finding that deserves to be mentioned is that, following chemotherapy, ApoE4 carriers are at increased risk of cognitive deficits (Ahles et al., 2003). In the same line, older ApoE4 carriers are at increased risk of delirium following surgery (Leung et al., 2007). The assessment of ApoE might therefore help guide the choice of treatment or give knowledge about potential side effects.

In addition to orienting treatment management, a better comprehension of disease interaction with gene variants may eventually help to create new treatments. A novel approach that is getting increasing attention is the potential conversion of ApoE4 into a molecule that is structurally and functionally similar to ApoE3 (or ApoE2). Recent studies that have investigated small molecules to block domain interaction in ApoE4, have largely been successful in preventing the deleterious effects associated with E4 (Mahley and Huang, 2012b; Liu et al., 2013). Other strategies, such as using drugs that promote ApoE levels, increase the ABC 1 expression, increase ApoE receptor 2, or restore brain vascular integrity might also be potential targets (Champagne et al., 2003; Bu, 2009; Liu et al., 2013). Cyclophilin A for instance seems to be a valuable target to offset the negative impact of ApoE4 on BBB and potentially improve, or reverse, microvascular, and cerebral blood flow reduction (Bell et al., 2012). Because neurovascular defects can initiate neuronal dysfunction and neurodegenerative changes, such treatment might be valuable to prevent or slow down neurodegeneration in older adults. As previously mentioned, preventing BBB breakdown might also have a positive effect on Aβ accumulation (Deane et al., 2008; Bell and Zlokovic, 2009). Even if research on these new therapeutic approaches is not yet accessible in humans, their potential therapeutic implications have already created enthusiasm in the scientific community (Bu, 2009; Ramaswamy and Kordower, 2011; Mahley and Huang, 2012a).

### ApoE, nutrigenetic, and lifestyle

Interestingly, while ApoE4 carriers are usually least sensitive to statins, they are highly responsive to reduced fat diets. A systematic review conducted in 2003 suggests that ApoE4 carriers tend to show the greatest decrease in LDL-cholesterol and total-cholesterol following dietary changes (Masson et al., 2003). Saito et al. reported similar results in patients with type 2 diabetes after a 14 day reduced fat diet therapy, showing a mean reduction of 15.6% for LDL-cholesterol and of 16.3% for total cholesterol in the ApoE3/4 group compared to 0.7 and 6.6%, respectively in the ApoE3/3 group (Saito et al., 2004). Variation in HDL-cholesterol following a diet rich in polyunsaturated fat is unclear (Masson et al., 2003; De Andrade et al., 2010); however, physical activity, particularly in ApoE4 carriers (Bernstein et al., 2002), increases HDL cholesterol (Marrugat et al., 1996; Kokkinos and Fernhall, 1999). These findings are encouraging because they suggest that alternative and/or complementary approaches can be use to control metabolic variation in ApoE4 carriers.

As is well known, the lipid profile of a person has important implications for CVD development. Controlling lipid levels might also have a beneficial effect for AD prevention. In a recent *in vivo* study it was shown that increased LDL cholesterol was associated with increased brain Aβ levels (Reed et al., 2014). Higher HDL cholesterol levels have also been found to be protective against atrophy in brain regions vulnerable to AD (Villeneuve et al., 2014). Therefore, knowing the ApoE genotype of a person might influence the type of treatment proposed, or at least highlights the importance of incorporating lifestyle changes as part of an intervention plan.

### ApoE genotype and compliance to treatment

In addition to the drug response dosage, the APOE genotype might influence adherence to treatment, a primary concern in medicine (Maitland-Van Der Zee et al., 2003). Indeed, in a population-based study, Maitland-van der Zee et al. found that subjects with ApoE4/4 genotype had a 2.28-fold increased risk of ending a statin therapy when compared to individuals with the ApoE3/3 genotype (Maitland-Van Der Zee et al., 2003). This risk was predominant among men. Unfortunately, the reasons for discontinuing statin were not explored, however adverse side effects is the principal reason for discontinuing antihyperlipidemic therapy (Andrade et al., 1995). ApoE carriers in the Maitland-Van Der Zee et al. study showed a trend toward higher dosage when compared to the other genotypes, which might have resulted in more severe side effects (Maitland-Van Der Zee et al., 2003). It is also possible that ApoE4 genotype is associated with more side effects independently of dosage as this has been found in other diseases (De La Fuente-Fernandez et al., 1999; Porrata-Doria et al., 2010). The second most frequent reason for ending therapy is therapeutic inefficacy (Andrade et al., 1995). Again, this reason might explain why ApoE4 is associated with an increased risk of drug discontinuation given the well-known lower response of ApoE4 carriers to treatment (Nieminen et al., 2008).

## Conclusions

This review gives evidence that ApoE genotype affects pre-symptomatic risk, diagnosis, prognosis, and treatment response for a variety of diseases. Despite these multiple associations, its inclusion in personalized medicine faces a major challenge because for most if not all diseases, ApoE is a risk and not a causal factor, and the many inconsistent findings in the literature suggest that ApoE-associated diseases are the result of complex interactions. Inclusion of ApoE in personalized medicine might be most effective as part of a broader panel of information. Likewise, the decision to assess ApoE genotype may best be made on a case-by-case basis.

Understanding the source of ApoE-interactions might yield great potential for disease prevention. It could be particularly important in patients with a family history of type III dysbetalipoproteinemia because by preventing other dyslipidemic factors it may be possible to postpone or stop symptomatic expression of the disease. Increasing evidence also suggests that improving one's lifestyle might help delay onset of AD and CVD. In such cases, one could however argue that good lifestyle habits should be promoted irrespective of genotype, and that assessing ApoE status would therefore not be needed. Even if this is correct, knowing that actions can be taken to modify a genetic risk is valuable, particularly in diseases such AD where no treatment is currently available.

In regard to treatment, ApoE status would probably not change the initial treatment plan, but this genetic information might help to guide recommendations for alternative treatments in cases of limited drug efficacy or adverse side effects. For instance, while ApoE4 carriers appear to be less responsive to lipid lowering therapy, they are more sensitive to diet and physical activity. Therefore, genotype specific treatment plans could be introduced as part of clinical care when a person displays suboptimal response to common treatments. ApoE genotype information could also be used to inform treatment in specific circumstances. For example, if a medical team is considering the pros and the cons of performing surgery in a frail older adult, knowing the patient's ApoE status could inform the decision. An ApoE4 carrier might be advised against the surgery. One area where ApoE should certainly be included is in clinical trials, particularly those concerning CVD and AD, as ApoE status clearly affects outcome. Following from this, ApoE could be used a target for new drug interventions given its broad range of action.

Ethical implications of ApoE genotyping should also be considered. For instance, what are the implications of disclosing that a person is an ApoE4/4 carrier? On one hand, the patient might be motivated to modify environmental factors that are within their control in order to reduce risk of associated diseases. On the other hand, the stress and potential adverse consequences caused by this information might be more detrimental than beneficial. There is not a large evidence base examining the psychological effects of disclosure, however in individuals with a familial history of AD, disclosure of their ApoE genotype did not increase distress for e4-carriers and indeed reduced it for non-e4 carriers (Green et al., 2009). It is also important to address whether genetic information would be available to health insurance companies, as this could profoundly affect access to care. Finally, one major concern raised about testing for ApoE genotype is that patients would be over-diagnosed and over-treated.

In conclusion, the effect of ApoE on disease risk is complex, however knowing the ApoE status of a person along with medical and family history, medication, and lifestyle could give valuable information for personalized medicine. More research into understanding the factors and mechanisms involved in ApoE-related interactions could improve awareness of disease susceptibility, prevention strategies, and guide person-centered therapeutic approaches.

### Conflict of interest statement

The authors declare that the research was conducted in the absence of any commercial or financial relationships that could be construed as a potential conflict of interest.
